# A mixed perturbative-nonperturbative treatment for strong light-matter interactions

**DOI:** 10.1515/nanoph-2023-0863

**Published:** 2024-02-01

**Authors:** Carlos J. Sánchez Martínez, Johannes Feist, Francisco J. García-Vidal

**Affiliations:** Departamento de Física Teórica de la Materia Condensada and Condensed Matter Physics Center (IFIMAC), Universidad Autónoma de Madrid, E-28049 Madrid, Spain; Institute of High Performance Computing (IHPC), Singapore 138632, Singapore

**Keywords:** quantum nanophotonics, subwavelength cavity-QED

## Abstract

The full information about the interaction between a quantum emitter and an arbitrary electromagnetic environment is encoded in the so-called spectral density. We present an approach for describing such interaction in any coupling regime, providing a Lindblad-like master equation for the emitter dynamics when coupled to a general nanophotonic structure. Our framework is based on the splitting of the spectral density into two terms. On the one hand, a spectral density responsible for the non-Markovian and strong-coupling-based dynamics of the quantum emitter. On the other hand, a residual spectral density including the remaining weak-coupling terms. The former is treated nonperturbatively with a collection of lossy interacting discrete modes whose parameters are determined by a fit to the original spectral density in a frequency region encompassing the quantum emitter transition frequencies. The latter is treated perturbatively under a Markovian approximation. We illustrate the power and validity of our approach through numerical simulations in three different setups, thus offering a variety of scenarios for a full test, including the ultra-strong coupling regime.

## 1 Introduction

In recent years, the development of nanophotonic devices where light is confined at length scales far below the optical wavelength is leading to new venues for integrated circuitry, optical quantum computing, solar and medical technologies [1]. The nanophotonic structures capable of obtaining such extreme light confinement are plasmonic (metallic) and hybrid metallodielectric nanocavities. In particular, the location of a quantum emitter in close proximity to such nanostructures results in promising enhanced light-matter interactions, ranging from the enhancement of the spontaneous emission rate [2], [3] (known as Purcell effect [4]) to the possibility of reaching strong [5], [6], [7], [8] and, even, ultra-strong light-matter coupling [9].

The complex geometry and the lossy character inherent in these metallic-based nanodevices define an arbitrary electromagnetic (EM) environment that is open, dispersive and absorbing. In this scenario, the EM mode spectrum is typically characterized by arbitrarily broad and overlapping resonances embedded in the continuum. The quantization of this medium-assisted EM field constitutes a genuine challenge as losses must be treated explicitly, such that traditional techniques of quantization fail [10]. These difficulties are formally solved by macroscopic quantum electrodynamics (QED) [11], [12]. This framework provides a quantization scheme for the EM field in arbitrary structures, including dispersive and lossy materials. The outcome is an EM field described through a four-dimensional continuum of quantum harmonic oscillators in real space and frequency. Despite the power and generality of this formalism, recently used for exploring the emerging phenomena in nanophotonics [13], [14], [15], [16], [17], a description based on an extremely large collection of modes like that represents a clear drawback. On the one hand, it restricts the direct applicability of this approach to cases where the EM modes can be treated perturbatively or eliminated by Laplace transform or similar techniques, and on the other hand, it precludes the desirable application of standard quantum optics (cavity QED) protocols based on a single or a few isolated modes interacting with a quantum emitter and capable of accounting for strong light-matter interactions. Several important steps towards making a connection with such practical quantum optics approaches have been taken in the last decades [18], [19], [20], and quantized few-mode descriptions for specific plasmonic geometries such as surfaces [21], spheres [22], [23], [24], [25] or sphere dimers [26], [27] have been obtained. However, until recently no general frameworks for achieving few-mode field quantization in arbitrary structures were available, particularly in the case of hybrid structures where it is necessary to describe modes with quite different characteristics and mutual coupling.

This was solved in the last few years with two complementary approaches, both building on the framework of macroscopic QED. One relies on using quasinormal modes [28], [29], which are open cavity modes with complex eigenfrequencies. They are constructed by combining different macroscopic QED modes, constituting a nonorthogonal basis that is then orthonormalized and treated with some approximations to arrive at a standard quantum optics Hamiltonian containing a few lossy discrete modes interacting with each other [30], [31]. The other approach [32] is inspired by tools from the field of open quantum systems [33], [34]. It replaces the original EM environment by a model system involving only a small number of lossy interacting discrete modes. The model permits the calculation of a compact closed expression for its spectral density, such that a fitting procedure to the original spectral density provides a few-mode field quantization of the EM field. In comparison with the previous quasinormal-mode expansion, this approach requires fewer modes for convergence. Furthermore, it has recently been extended to the treatment of both multiple emitters [35] and the ultra-strong coupling regime [36]. Its main downside is that, depending on the complexity of the EM environment, the number of discrete modes required for an accurate fit can still be larger than ideally wished to ensure low computational cost.

In the present work, we tackle this issue. We explore an approach capable of reducing the number of modes needed in [32] and based on exploiting the underlying physics of the interaction. We divide the spectral density into two contributions in order to separate effectively the part of the EM environment strongly coupled to the emitter from that one which is weakly coupled to it. The strongly coupled environment, which induces non-Markovian dynamics, is treated nonperturbatively using the technique developed in [32] of finding an auxiliary few-mode model for such environment. The residual environment is instead treated perturbatively under the assumption of Markovianity, reflecting its effect in an energy shift on the emitter energy levels dubbed Casimir-Polder (CP) energy shift [37]. Note that this mixed treatment avoids the use of discrete modes for the part of the environment that is treated perturbatively.

We demonstrate that our model allows the description of the emitter dynamics through a Lindblad-like master equation, even for the ultra-strong coupling regime in which it is well-known that standard Lindblad dissipation terms give rise to unphysical effects. We then test our model validity through numerical calculations of the population of a two-level emitter in the problem of spontaneous emission, in three different setups. The first two exhibit light-matter interactions in the strong coupling regime: one is a canonical test example consisting of a Lorentzian-like spectral density, and the other one is a realistic hybrid metallodielectric nanostructure. The third setup goes beyond exhibiting a real ultra-strong coupling case.

## 2 Model

Our starting point is the general Hamiltonian describing a quantum emitter linearly coupled to a collection of bosonic modes representing the medium-assisted EM field:

(1) $$H=H_{e}+\underset{\alpha }{\sum }\omega_{\alpha }a_{\alpha }^{†}a_{\alpha }+D_{e}\underset{\alpha }{\sum }\left(g_{\alpha }a_{\alpha }+\text{h.c.}\right),$$

where we here and in the following set ℏ = 1. The emitter is described by its Hamiltonian *H*_*e*_ and dipole operator ${\vec{D}}_{e}=D_{e}d\vec{n}$ , where all transitions are assumed to be oriented along the same direction $\vec{n}$ , and *d* is a characteristic dipole moment such that *D*_*e*_ is unitless. The EM modes are described by their annihilation operators *a*_*α*_, frequencies *ω*_*α*_, and coupling to the emitter *g*_*α*_ (which depends on $\vec{n}$ and *d*). The full information about the light-matter coupling is then encoded in the so-called spectral density:

(2) $$J\left(\omega \right)=\underset{\alpha }{\sum }|g_{\alpha }{|}^{2}\delta \left(\omega -\omega_{\alpha }\right).$$

Although our approach is valid for multi-level emitters, from now on we will consider a two-level system (TLS) with ground state |*g*⟩, excited state |*e*⟩ and transition energy *ω*_*e*_. Under this approximation, the emitter operators become *H*_*e*_ = *ω*_*e*_ *σ* ^+^ *σ* ^−^ and *D*_*e*_ = *σ* ^+^ + *σ* ^−^, with ladder operators *σ* ^+^ = |*e*⟩⟨*g*| and *σ* ^−^ = |*g*⟩⟨*e*|.

We note that the index *α* in Eq. (1) and Eq. (2) is a compact notation to represent a set of both discrete and continuous variables (for which the sum becomes an integral). In particular, within macroscopic QED, *α* represents a combined index for four continuous (three spatial and one frequency) and two discrete (Cartesian direction and electric or magnetic excitation) degrees of freedom [12]. The Hamiltonian in Eq. (1) then describes the physical system we are interested in: a quantum emitter interacting with the EM field supported by a nanophotonic structure, described within the Power-Zienau-Woolley picture [38] and the long-wavelength (or dipole) approximation. The spectral density Eq. (2) can then be written in terms of the classical dyadic EM Green’s tensor $G\left(\vec{r},{\vec{r}}^{\prime },\omega \right)$ [37], [39]:

(3) $$J\left(\omega \right)=\frac{d^{2}\omega^{2}}{\pi \varepsilon_{0}c^{2}}\vec{n}\cdot ImG\left({\vec{r}}_{e},{\vec{r}}_{e},\omega \right)\cdot \vec{n},$$

where ${\vec{r}}_{e}$ is the emitter position. The Green’s tensor of Maxwell’s equations [12] fulfills

(4) $$\left[\nabla \times \frac{1}{\mu \left(\vec{r},\omega \right)}\nabla \times -\frac{\omega^{2}}{c^{2}}\varepsilon \left(\vec{r},\omega \right)\right]G\left(\vec{r},{\vec{r}}^{\prime },\omega \right)=\delta \left(\vec{r}-{\vec{r}}^{\prime }\right),$$

where $\delta \left(\vec{r}-{\vec{r}}^{\prime }\right)$ is the Dirac-delta tensor and $\varepsilon \left(\vec{r},\omega \right)$ and $\mu \left(\vec{r},\omega \right)$ are, respectively, the electric permittivity and magnetic permeability accounting for our electromagnetic configuration. Note that in free-space (*ɛ* = *μ* = 1), the solution of Eq. (4) is analytical:

(5) $$G_{0}\left(\vec{r},{\vec{r}}^{\prime },\omega \right)=\left[I+\frac{1}{k^{2}}\nabla ⊗\nabla \right]\frac{e^{\mathrm{ikR}}}{4\pi R},$$

where **I** is the identity tensor, $R=|\vec{r}-{\vec{r}}^{\prime }|$ and *k* = *ω*/*c*. In the presence of a nanostructure, the solution of Eq. (4) is generally no longer analytical but can be written as **G** = **G**_0_ + **G**_*s*_, where **G**_*s*_ accounts for the fields scattered by the nanostructure. Similarly, the spectral density can be split as *J*(*ω*) = *J*_0_(*ω*) + *J*_*s*_(*ω*) provided by Eq. (3):

(6a) $$J_{0}\left(\omega \right)=\frac{d^{2}\omega^{3}}{6\pi^{2}\varepsilon_{0}c^{3}},$$

(6b) $$J_{s}\left(\omega \right)=\frac{d^{2}\omega^{2}}{\pi \varepsilon_{0}c^{2}}\vec{n}\cdot ImG_{s}\left({\vec{r}}_{e},{\vec{r}}_{e},\omega \right)\cdot \vec{n},$$

where in Eq. (6a) we have used that the free-space Green’s tensor fulfills $\vec{n}\cdot ImG_{0}\left({\vec{r}}_{e},{\vec{r}}_{e},\omega \right)\cdot \vec{n}=\frac{\omega }{6\pi c}$ .

**Figure 1:**
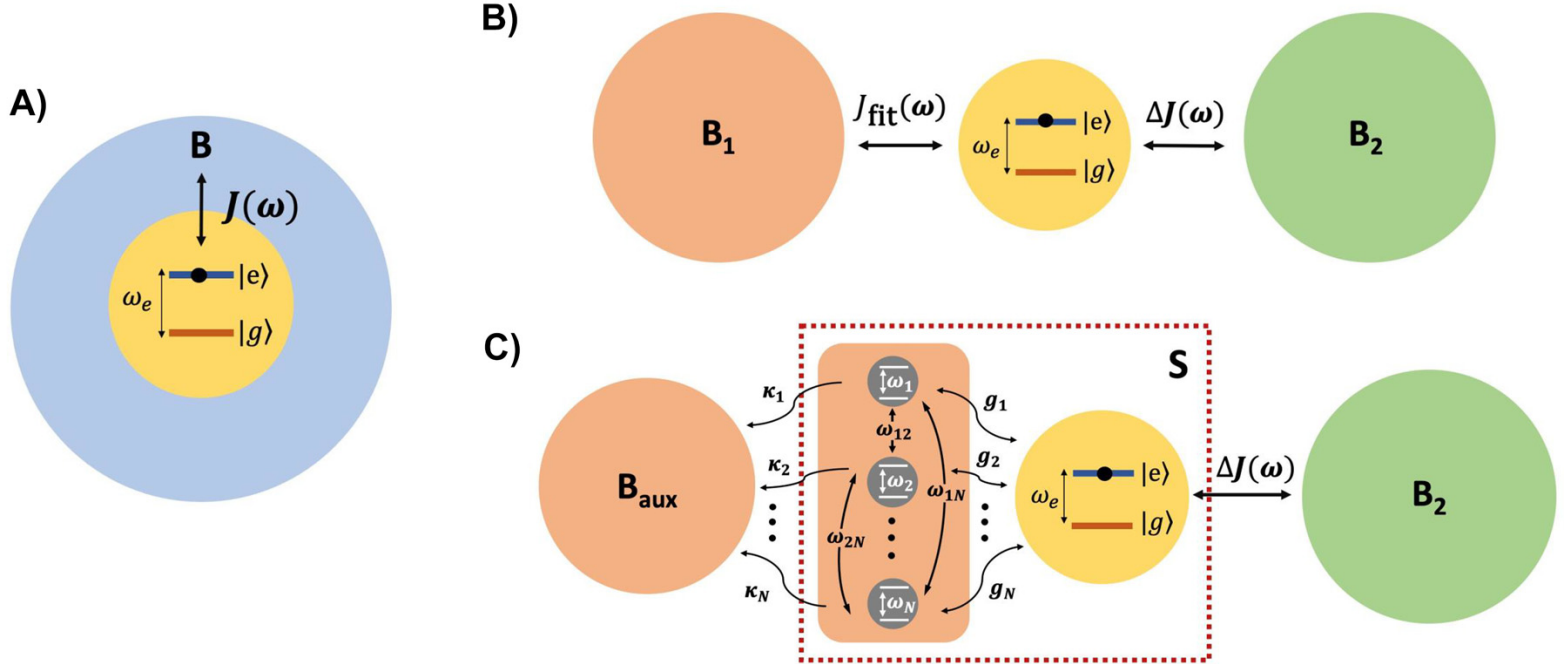
Graphical representation of our composite system. A) Original configuration: Emitter interacting with a EM bath *B* through the spectral density *J*(*ω*). B) Original configuration with the EM bath *B* split into two independent contributions, *B*_1_ and *B*_2_, to which the emitter coupling is encoded in the spectral densities *J*_fit_(*ω*) and Δ*J*(*ω*), respectively. C) Model configuration: *B*_1_ is substituted by *N* interacting modes coupled to a spectrally flat auxiliary EM bath *B*_aux_. The discrete modes are also coupled to the emitter, conforming a new open quantum system *S*.

The above reflects a more general property: the spectral density can be rearranged arbitrarily and written as the sum of different contributions that can be treated independently, with only their sum being physically meaningful. This can also be understood from Eq. (2), where the sum over modes *α* can be obviously split into several sums over arbitrary groups of indices *α*. We exploit this freedom to write the spectral density as the sum of two contributions, *J*(*ω*) = *J*_fit_(*ω*) + Δ*J*(*ω*). The first, *J*_fit_(*ω*), describes modes close to resonance with the emitter that can lead to non-Markovian effects such as strong coupling, while the second, Δ*J*(*ω*), describes small and/or off-resonant contributions that can be treated perturbatively. Within this picture, the emitter is coupled to two independent EM baths *B*_1_ and *B*_2_ described by *J*_fit_(*ω*) and Δ*J*(*ω*), respectively (see Figure 1A and B).

The light-matter interaction described by Δ*J*(*ω*) can be then directly treated through a perturbative approach and within the Markov approximation. This approximation should be valid as long as Δ*J*(*ω*) is small and flat enough over the bandwidth of frequencies that the emitter is resonant with. On the contrary, the light-matter interaction with *B*_1_, described by *J*_fit_(*ω*) and characterized by nonperturbative features, is addressed following the strategy presented in [32]: we replace *B*_1_ by an equivalent environment consisting of *N* interacting discrete modes coupled to a fully Markovian bath *B*_aux_. In the resulting model configuration (see Figure 1C), the bipartite system *S* (emitter + discrete modes) is considered as the open quantum system, with Hamiltonian:

(7) $$H_{S}=H_{e}+\overset{N}{\underset{i,j}{\sum }}\omega_{ij}a_{i}^{†}a_{j}+\overset{N}{\underset{i}{\sum }}g_{i}\left(\sigma^{+}+\sigma^{-}\right)\left(a_{i}+a_{i}^{†}\right),$$

where *ω*_*ij*_ encodes the mode energies and couplings, *κ*_*i*_ their decay rates, and *g*_*i*_ their coupling to the emitter (including the transition dipole moment *d*). The value of these parameters is obtained by a nonlinear fit of *J*_fit_ to the desired region of *J*. This fitting can be performed relatively straightforwardly as *J*_fit_ is given by a compact expression:

(8) $$J_{\mathrm{fi}t}\left(\omega \right)=\frac{1}{\pi }\vec{g}\cdot Im\left[\frac{1}{\tilde{H}-\omega }\right]\cdot {\vec{g}}^{\text{T}},$$

with $\vec{g}=\left(g_{1},g_{2},\ldots ,g_{N}\right)$ and ${\tilde{H}}_{ij}=\omega_{ij}-\frac{i}{2}\kappa_{i}\delta_{ij}$ . After choosing an initial guess of the parameter values, they are optimized with standard methods of nonlinear fitting to find values that minimize the difference between the physical and the fitted spectral density. Note that the results are not unambiguous, as different sets of parameters can give very similar (or even identical) spectral densities [40]. Furthermore, the number of modes required to achieve a good fit depends on the complexity of the spectral density and is a manually chosen “hyperparameter”. While its minimum value is determined by the number of resonances within the fitted window, it can be increased to improve the quality of the fit as required. Note that the number of modes is in this sense not a physically meaningful quantity (only the spectral density is), but a computational parameter that can be chosen to achieve a desired accuracy.

The coupling of *S* with the two baths is then treated perturbatively following the standard Markovian procedure in open quantum systems textbooks [33], [34] (which is exact for *B*_aux_ [41] and approximate for *B*_2_). This leads to a Lindblad master equation for the dynamics of the system *S*:

(9) $$\begin{array}{ll} \frac{\text{d}}{dt}\rho_{S}\left(t\right) & =-i\left[H_{S}+H_{\text{CP}},\rho_{S}\left(t\right)\right]+\gamma_{mod}D_{\sigma^{-}}\left[\rho_{S}\left(t\right)\right]\\ & \;+\overset{N}{\underset{i}{\sum }}\kappa_{i}\;\;D_{a_{i}}\left[\rho_{S}\left(t\right)\right], \end{array}$$

where $D_{o}\left[\rho_{S}\left(t\right)\right]=o\rho_{S}\left(t\right)o^{†}-\frac{1}{2}\left\{o^{†}o,\rho_{S}\left(t\right)\right\}$ is a standard Lindblad dissipator, *H*_CP_ = −Δ_mod_ *σ* ^+^ *σ* ^−^ encodes the CP energy shift Δ_mod_, and *γ*_mod_ is a decay rate. Both parameters, Δ_mod_ and *γ*_mod_, arise from the perturbative treatment of Δ*J* within an additional rotating wave approximation for the light-matter coupling, and are given by

(10a) $$\Delta_{mod}=P\overset{\infty }{\underset{-\infty }{\int }}\text{d}\omega \frac{\Delta J_{s}\left(\omega \right)}{\omega -\omega_{e}},$$

(10b) $$\gamma_{mod}=2\pi \Delta J\left(\omega_{e}\right),$$

where Δ*J*_*s*_(*ω*) = *J*_*s*_(*ω*) − *J*_fit_(*ω*) and P indicates a principal value integral. Here, *J*_*s*_ instead of the full spectral density appears as the energy shift, since the free-space contribution *J*_0_ leads to a diverging shift when inserted directly in Eq. (10a), but gives the small free-space Lamb shift when treated correctly [37], [42]. It is thus assumed that its influence is already included in the emitter transition frequency *ω*_*e*_. Note also that the integral over frequencies in Eq. (10a) extends over the full real axis. While *J*(*ω*) is nonzero only for positive frequencies (at zero temperature, as considered throughout this manuscript), *J*_fit_(*ω*) is defined and non-zero for all frequencies.

The approach described above has two potential advantages compared to the one in [32] that it extends: first, it can be used to reduce the number of auxiliary coupled oscillators that have to be included in *S* by only fitting a reduced part of the spectrum, and second, it can be used to mitigate any inaccuracies in the fit by including the resulting correction Δ*J*(*ω*) = *J*(*ω*) − *J*_fit_(*ω*) in the master equation, albeit only within the Markovian approximation. Below, we investigate the accuracy of the resulting model in different scenarios. It can be anticipated that the model will work well if those parts of the spectral density that induce non-Markovian dynamics on the emitter are well-described by the auxiliary model described by *J*_fit_(*ω*), which usually requires that the fit is accurate at least within a spectral window close to the emitter transition frequency. We note that if the whole light-matter interaction is in the weak-coupling regime, it can be treated fully perturbatively, and the model is not needed. This case is equivalent to setting *J*_fit_(*ω*) = 0.

For the case of interest where the overall coupling is non-Markovian, a criterion to estimate the validity of the splitting can be formulated by utilizing that the enforced good agreement between *J*(*ω*) and *J*_fit_(*ω*) in the spectral region close to the emitter frequency implies that Δ*J*(*ω*) is small within that region (and presumably larger outside), so that it naturally splits into the two regions *ω* < *ω*_*e*_ and *ω* > *ω*_*e*_. The validity of the perturbative treatment of Δ*J*(*ω*) can then be checked by treating each of the two regions separately and veryifying that the resulting interaction is indeed perturbative. This can be done by, for example, checking that the “reaction mode” [40], [43], [44] that subsumes the bath-emitter interaction in each region is perturbatively coupled to the emitter. This leads to the condition $\beta_{\pm }=\frac{g_{\pm }^{2}}{{\left(\omega_{\pm }-\omega_{e}\right)}^{2}}\ll 1$ . Here, the effective coupling is given by $g_{\pm }^{2}=\int_{A_{\pm }}\Delta J\left(\omega \right)d\omega$ and the effective frequency by $\omega_{\pm }=\int_{A_{\pm }}\omega \Delta J\left(\omega \right)d\omega /\int_{A_{\pm }}\Delta J\left(\omega \right)d\omega$ , where *A*_+_ (*A*_−_) is the region of frequencies where *ω* > *ω*_*e*_ (*ω* < *ω*_*e*_). In the examples shown below, the condition *β*_±_ ≪ 1 is always fulfilled.

The above definition of Δ*J*(*ω*) as the difference between the physical and the fitted spectral density unveils a subtle point: while both *J*(*ω*) and *J*_fit_(*ω*) correspond to the spectral density of physical systems and are thus strictly non-negative functions, Δ*J*(*ω*) does not necessarily fulfill this constraint. This does not present a particular problem for the CP-shift Δ_mod_, which in any case can be a positive or negative energy shift, but can appear problematic for the decay rate *γ*_mod_, since the Lindblad master equation is not a completely positive map if *γ*_mod_ < 0, and the resulting terms do not describe decay, but “anti-decay”, i.e., an exponential growth of population.1Note that is not the same as a “pumping” Lindblad term, which corresponds to a normal Lindblad term with positive rate and an associated operator that lifts the system to a state with higher energy (e.g., $\Gamma_{\text{pump}}D_{\sigma^{+}}\left[\rho \right]$ ). Note that in principle, the derivation of the Lindblad master equation requires that the spectral density be positive, and allowing for negative rates is thus not strictly justified. In this sense, anti-decay terms are a generalization of existing results to a regime outside their original range of validity. In the context of open quantum systems, similar generalizations are commonly done with negative-frequency harmonic oscillators, which are not eigenstates of a physical potential, but can be useful tools to generalize approaches to new regimes [45].

We will show below that the appearance of negative rates is not an issue in practice when the description is sufficiently accurate. This is consistent with similar results found for the Bloch-Redfield approach [46], i.e., a perturbative treatment of a bath within the Born-Markov approximation, which can induce negative decay rates if no additional secular approximation is performed [34]. For most cases we study, the problem does not appear, since the spectral density is fitted accurately close to the emitter frequency and thus *γ*_mod_ = 2*π*Δ*J*(*ω*_*e*_) ≈ 0. However, in subsection 3.3 we treat a system in the ultra-strong coupling regime where counterrotating terms in the light-matter interaction cannot be neglected. For this system, we show that a negative-rate “anti-Lindblad” term can efficiently cancel unphysical artificial pumping effects that otherwise appear [36]. This term arises naturally from the perturbative treatment of Δ*J* when, unlike Eq. (9), the rotating-wave approximation in the light-matter coupling is not performed, such that the resulting generalized Lindblad-like master equation is

(11) $$\begin{array}{ll} \frac{\text{d}}{dt}\rho_{S}\left(t\right) & =-i\left[H_{S}+H_{\text{CP}}+{\tilde{H}}_{\text{CP}},\rho_{S}\left(t\right)\right]+\gamma_{mod}D_{\sigma^{-}}\left[\rho_{S}\left(t\right)\right]\\ & \;+{\tilde{\gamma }}_{mod}D_{\sigma^{+}}\left[\rho_{S}\left(t\right)\right]\;+\;\sum_{i}^{N}\kappa_{i}D_{a_{i}}\left[\rho_{S}\left(t\right)\right], \end{array}$$

which contains both an extra CP term ${\tilde{H}}_{\text{CP}}=-{\tilde{\Delta }}_{mod}\sigma^{+}\sigma^{-}$ , where

(12) $${\tilde{\Delta }}_{mod}=-P\overset{\infty }{\underset{-\infty }{\int }}\text{d}\omega \;\frac{\Delta J_{s}\left(\omega \right)}{\omega +\omega_{e}},$$

and the additional Lindblad term with rate ${\tilde{\gamma }}_{mod}=2\pi \Delta J\left(-\omega_{e}\right)$ . This rate is always negative since *J*(*ω*) = 0 for negative frequencies *ω* < 0, while *J*_fit_(*ω*) ≥ 0 for any *ω*, such that the term becomes an “anti-Lindblad” one as described above. Observe as well that Eq. (12) is identical to Eq. (10a) performing the substitution *ω*_*e*_ → −*ω*_*e*_ (the overall minus sign is a matter of convention to write both CP energy terms preserving the same form).

## 3 Results

We test the accuracy and regime of validity of our model by performing numerical simulations of the excited-state population of a TLS, $\left\langle \sigma^{+}\sigma^{-}\right\rangle \left(t\right)$ , for the paradigmatic problem of spontaneous emission. Notice that our model allows the computation of expectation values of any observable *O*, $\left\langle O\right\rangle \left(t\right)=\mathrm{Tr}\left\{O\rho_{S}\left(t\right)\right\}$ , since it provides the density matrix operator *ρ*_*S*_(*t*). Furthermore, it is not restricted to the single-excitation subspace [35], [36].

We consider three different model EM environments: the first corresponds to a simple model where the spectral density is described by a sum of Lorentzian resonances, the second one is a realistic hybrid metallodielectric nanostructure, and the third one is a single-mode setup corresponding to a two-level emitter under ultrastrong coupling to a single physical mode. In all three systems, the light-matter coupling is strong enough to obtain non-Markovian effects, as the weak (Markovian) coupling regime can already be described accurately by fully perturbative approaches (equivalent to setting *J*_fit_(*ω*) = 0 in our model). The first two systems are treated within the rotating-wave approximation and described by Eq. (9), while the third one is within the ultra-strong coupling regime where this approximation is not valid and the effective master equation is given by Eq. (11). In all cases, we compare the results obtained with our approach with an exact solution obtained by direct discretization of the original Hamiltonian in Eq. (1) (which is numerically feasible for propagation over short times and when no decoherence apart from that induced by the bath is present, such that the dynamics is purely coherent).

### 3.1 Lorentzian model spectral density

We start with a test case consisting of an example EM environment characterized by a spectral density that is the sum of Lorentzian resonances, $J_{1}\left(\omega \right)=\sum_{i}\frac{g_{i}^{2}}{\pi }\frac{\kappa_{i}/2}{{\left(\omega -\omega_{i}\right)}^{2}+{\left(\kappa_{i}/2\right)}^{2}}$ , which corresponds to the non-interacting limit of Eq. (8) (*ω*_*ij*_ = 0 for *i* ≠ *j*). The non-interacting character of the modes and the flexibility for tuning their strength *g*_*i*_, width *κ*_*i*_, and frequencies *ω*_*i*_ offer an ideal scenario for gaining intuition on the model. In particular, since the form is exactly that of *J*_fit_(*ω*), the splitting into *J*_fit_(*ω*) and Δ*J*(*ω*) can be performed by just including some of the sum terms in *J*_fit_ without the need to perform any fitting. We use a 5-mode spectral density, considering two different situations: in the first case, $J_{1}^{a}\left(\omega \right)$ , the five peaks are spectrally well-separated, with a regular spacing of *ω*_*i*+1_ − *ω*_*i*_ = 0.6 eV, while in the second case, $J_{1}^{b}\left(\omega \right)$ , the separation between the peaks is reduced by half. In each configuration we study the scenario where the five modes fulfill the condition *g*_*i*_/*κ*_*i*_ > 1, guaranteeing the strong coupling regime (as will below reflected in a reversible dynamics). Note that this regime leads to the formation of hybrid light-matter states called polaritons (eigenstates of *H*_*S*_) whose frequencies determine the actual dynamics, and which are, in general, different from the frequencies in the uncoupled Hamiltonian.

**Figure 2:**
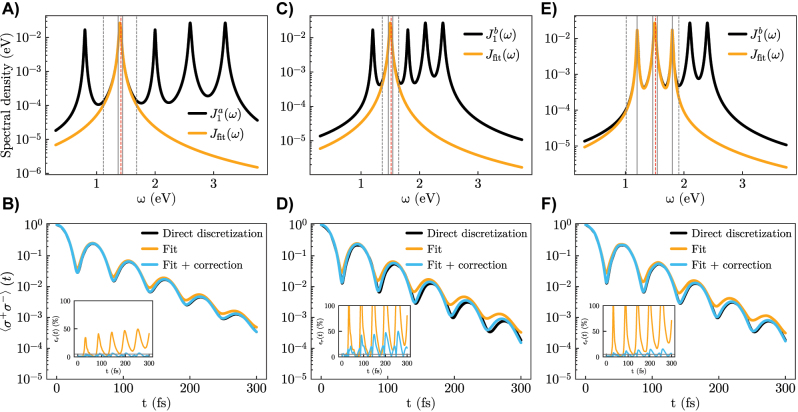
Numerical simulations on the test Lorentzian-like spectral density. Left column (A and B): Case with well-separated resonances. A) Spectral density splitting with a 1-mode model for *J*_fit_. The residual spectral density is not shown. The emitter transition frequency is indicated with a dashed red line and the fit range is comprised between the two dashed gray lines. The solid gray lines indicate the energy positions of the eigenstates of *H*_*S*_. B) Emitter excited-state population calculated from three different approaches: direct discretization (black), only considering *J*_fit_ (orange) and our full model (blue). The inset shows the relative error with respect to the exact result, where a solid gray horizontal line is traced at 5 %. Central column (C and D): The same as left column but for a squeezed spectral density. Right column (E and F): The same as central column but with a 3-mode model for *J*_fit_. Parameters: (A and B) *ω*_*e*_ = 1.4155, Δ_mod_ = 0.0021, *γ*_mod_ ≈ 0 [eV]; (C and D) *ω*_*e*_ = 1.5135, Δ_mod_ = 0.0043, *γ*_mod_ ≈ 0 [eV]; (E and F) *ω*_*e*_ = 1.5135, Δ_mod_ = 0.0041, *γ*_mod_ ≈ 0 [eV].

First, we focus on the results for the configuration with well-separated resonances, presented in Figure 2A and B, with the emitter resonant with the second mode. We use the simplest choice for *J*_fit_: a 1-mode model, treating nonperturbatively only the closest resonance to the emitter transition frequency. The rest of the spectral density is treated perturbatively through Δ*J*. The splitting of the spectral density is displayed in Figure 2A indicating also the emitter transition frequency (dashed red line), the range over which the fitted spectral density is accurate (dashed gray lines), and the energies of the formed polaritons (solid gray lines). The results of time propagation (see Figure 2B) show clearly that the dynamics is produced much more accurately by our model (blue lines) than by the use of only *J*_fit_ while ignoring the perturbative correction due to Δ*J*(*ω*) (orange lines). This is corroborated by the inset, which shows that the relative error, $\epsilon_{r}\left(t\right)=|\left\langle \sigma^{+}\sigma^{-}\right\rangle -{\left\langle \sigma^{+}\sigma^{-}\right\rangle }_{\text{exact}}|/{\left\langle \sigma^{+}\sigma^{-}\right\rangle }_{\text{exact}}$ , stays below about 5 % for the entire dynamics, while it reaches 50 % when only *J*_fit_ is used.

The results of reducing the spacing between resonances (spectral density $J_{1}^{b}\left(\omega \right)$ ) are presented in Figure 2C and D. Using a single-mode model as in the previous configuration now presents much larger deviations from the exact results. The reason for this is that the energies of the two polaritons formed by strong coupling between the emitter and the resonant mode are now much closer to the two nearest-non-fitted resonances, such that these two resonances also influence the emitter dynamics in a nonperturbative way that cannot be reflected in the CP energy shift, leading to multimode strong coupling effects. Including the two closest additional resonances in *J*_fit_ leads to a 3-mode model, with results displayed in Figure 2E and F. As could be expected, our approach now again works very well, highlighting the necessity of including a sufficiently wide frequency range in the fitted spectral density.

This first test example thus provides significant insight on the mixed perturbative-nonperturbative approach. First, we can deduce that the main requisite for its success is the inclusion in *J*_fit_ of all the spectral density contributions that lead to non-Markovian and strong-coupling effects and that cannot be captured accurately through a perturbative procedure. Second, even if this identification and fit is performed accurately, the role of the perturbative energy shift is fundamental to achieve an accurate description when the spectral density is non-negligible outside the fitted region. This demonstrates that the original goal can indeed be achieved: the number of discrete modes that must be included in *J*_fit_ to obtain accurate results can be significantly reduced compared to the case where *J*_fit_ is used for describing the whole spectral density.

### 3.2 Realistic nanostructure

**Figure 3:**
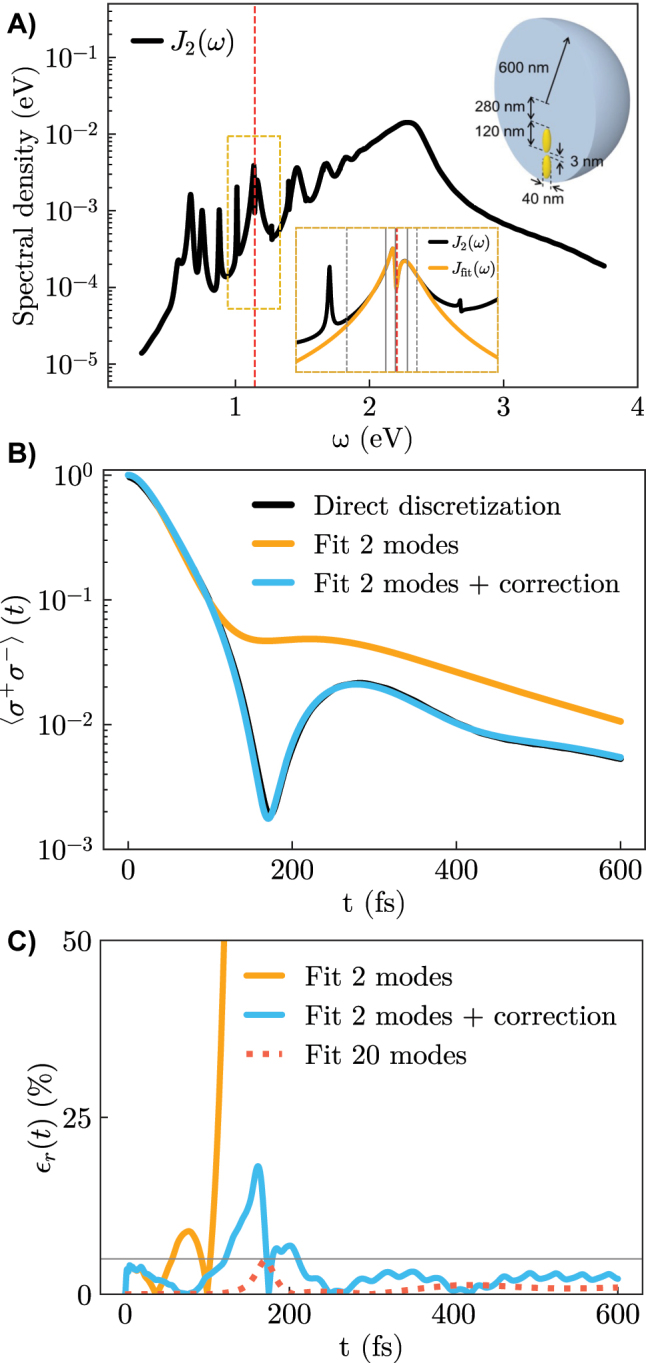
Numerical simulations on the hybrid metallodielectric nanostructure. A) Spectral density of the system. The upper right inset displays a sketch of the metallodielectric nanostructure. The bottom inset zooms in the 2-mode spectral density splitting in the region close to the emitter transition frequency. B and C) The same as the second row in Figure 2 but displaying the relative error in a separated figure (C). The dotted red line indicates the relative error obtained in [32] reproducing the whole spectral density with a 20-mode fit. Parameters: *ω*_*e*_ = 1.1445, Δ_mod_ = 0.0093, *γ*_mod_ ≈ 0 [eV].

These notions are confirmed with the study of the same realistic hybrid metallodielectric nanostructure treated in [32]. It consists of a dielectric GaP microsphere of radius 600 nm embedding two 120 nm long silver nanorods separated by a 3 nm gap and substantially displaced from the center of the sphere (see the upper right inset in Figure 3A). The emitter is located in the center of the gap, with parameters chosen to represent InAs/InGaAs quantum dots [47], with transition energy *ω*_*e*_ = 1.1445 eV and transition dipole moment *d* = 0.55 e nm. The hybrid nature of this structure results in a more complex spectral density *J*_2_(*ω*). It is characterized by Fano-like profiles that indicate interference effects between the different modes supported by the microsphere and the nanorods (see Figure 3A).

This complex spectral density is represented in [32] in a fully nonperturbative approach through a fit using 20 interacting modes. To illustrate the power of our approach, we use a 2-mode model for *J*_fit_, including only the two interacting modes close to resonance with the emitter (see the bottom inset in Figure 3A). As shown in Figure 3B and C, this is enough to obtain a reliable description of the emitter dynamics, with a relative error typically on the few-percent level. We note that the large maximum observed in the relative error close to *t* = 200 fs is a consequence of the small value of the population at that point.

The choice of this minimal model for *J*_fit_ is inspired by the fact that, although the light-matter interaction in this setup is strong (see the clear reversible behavior close to *t* = 200 fs), most of the modes do not in fact enter the strong coupling with the emitter, but instead only contribute an additional effective energy shift. This example is thus a clear demonstration of the power of our model when the frequencies that are on resonance with the emitter are correctly identified and the perturbative procedure can be safely performed, resulting in a large reduction of the number of discrete modes required.

### 3.3 Extension to the ultra-strong coupling regime

We finally illustrate the power of our model in the ultra-strong coupling regime. We study the same setup analyzed in [36]. It consists of a physically allowed extension of the quantum Rabi model, and is described by a spectral density corresponding to a single harmonic oscillator with frequency *ω*_*c*_ coupled to an Ohmic “background” bath. The resulting spectral density can be written as:

(13) $$J_{3}\left(\omega \right)=\theta \left(\omega \right)\frac{2g^{2}}{\kappa }\frac{\kappa \omega_{c}\omega }{{\left(\omega^{2}-\omega_{c}^{2}\right)}^{2}+\kappa^{2}\omega^{2}},$$

where we use the same parameters as in [36]: *ω*_*c*_ = *ω*_*e*_ = 0.58 meV, *g* = 0.25 meV and *κ* = 0.1 meV, which are typical for Landau polaritons formed in semiconductor quantum wells in the ultra-strong coupling regime [48], [49], [50]. Here, *g* represents the coupling between the emitter and the mode, and *κ* the losses of the mode. We note that this spectral density, as any physical spectral density, is non-zero only for positive frequencies.

**Figure 4:**
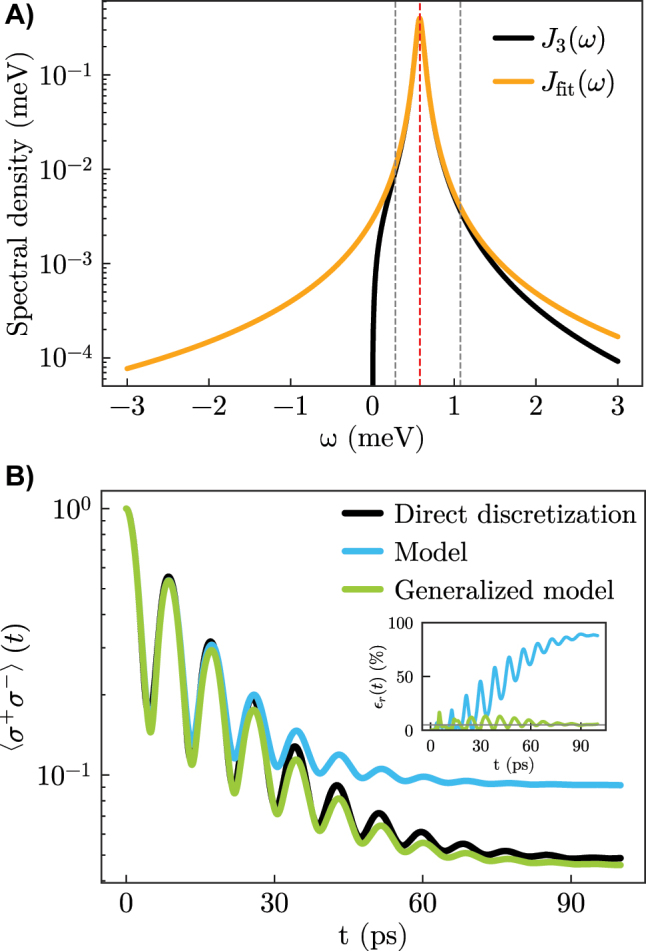
Numerical simulations on the single-mode setup supporting ultra-strong coupling effects. A) Spectral density splitting with a 1-mode model for *J*_fit_. B) Emitter population: the blue line is computed through Eq. (9), while the green line results from the generalized Eq. (11). The absolute relative error associated with the two models is displayed in the inset. Parameters: *ω*_*e*_ = 0.58, Δ_mod_ = 0.0026, ${\tilde{\Delta }}_{mod}=0.0026$ , *γ*_mod_ ≈ 0, ${\tilde{\gamma }}_{mod}=-0.0046$ [meV].

The results obtained using a 1-mode *J*_fit_ are presented in Figure 4A and B. Note that *J*_fit_ extends to negative frequencies, see Figure 4A. This cannot be avoided for a single-mode fit. While the flexibility of the coupled-oscillator model can be exploited to suppress these negative-frequency contributions [36], this requires the use of several additional auxiliary oscillators. When only a single mode is used for the fit and no perturbative corrections are performed, the presence of the negative-frequency components leads to artificial pumping effects, resulting in an unphysically large emitter population at later times. This is reflected in the results of Figure 4B, where the blue line shows the results obtained with Eq. (9), with a severely overestimated population, in particular in the steady state (reached at around 90 ps). However, when the perturbative corrections are included as described in the theory section, Eq. (11), the presence of the “anti-Lindblad” term with associated negative rate ${\tilde{\gamma }}_{mod}$ cancels the unphysical pumping effects, and the results (green line) are much closer to the exact ones (black line) at essentially the same numerical cost as the single-mode model. As the inset shows, the relative error within this method stays low for the whole dynamics, and the steady-state population is reproduced reasonably well.

## 4 Conclusions

We have presented an approach for describing light-matter interactions in arbitrarily complex nanophotonic systems in any coupling regime by using a mixed perturbative-nonperturbative description extending our previously developed few-mode quantization [32]. The approach is based on a splitting of the spectral density, *J*(*ω*), in order to effectively separate the part responsible for the non-Markovian and strong-coupling-based emitter dynamics, *J*_fit_(*ω*), from that which can be treated as a perturbation, Δ*J*(*ω*). The former is represented by a minimal collection of lossy interacting discrete modes coupled to fully Markovian background baths, while the latter is treated perturbatively with standard open quantum systems theory, leading to an energy shift on the emitter energy levels and additional Lindblad dissipator terms (which can contain negative dissipation rates). All this information is encoded in a compact simple Lindblad master equation.

We have tested our methods by calculating the population dynamics of an initially excited TLS in three different EM environments of varying complexity, investigating the strong and ultra-strong coupling regimes. We find that our model works accurately as long as *J*_fit_ is accurate over a sufficiently large frequency range to capture all non-Markovian effects. This condition can be fulfilled by identifying the spectral density region directly coupled to the relevant transitions frequencies of the system. The remaining spectral density can then be safely treated perturbatively. As a result, the final model achieves an accurate description with a significantly reduced numerical cost compared to the full model fitting the spectral density over its whole bandwidth.
